# Hypopharyngeal lipomatous hamartoma in piriform fossa: A case report and review of literature

**DOI:** 10.1016/j.jtumed.2022.08.011

**Published:** 2022-09-16

**Authors:** Abdulaziz A. Alsalem, Mohammed A. Alessa, Khaled A. Almanea, Bader A. Almanea, Abdullatif Khan

**Affiliations:** a Division of Otorhinolaryngology-Head and Neck Surgery, Department of Surgery, King Abdulaziz Medical City, Riyadh, Saudi Arabia; b King Abdullah International Medical Research Center, Riyadh, Saudi Arabia; c Department of Otolaryngology-Head and Neck Surgery, College of Medicine, King Saud University, Riyadh, Saudi Arabia; d Department of Otolaryngology-Head and Neck Surgery, Prince Sultan Military Medical City, Riyadh, Saudi Arabia; e College of Medicine, Shaqra University, Shaqra, Saudi Arabia; f Department of Pathology and Laboratory Medicine, King Abdullah Medical City, Riyadh, Saudi Arabia

**Keywords:** البلعوم السفلي, شحمي, دموي, الحفرة, الكمثرية, Fossa, Hamartoma, Hypopharynx, Lipomatous, Piriform

## Abstract

Most hypopharyngeal disease presentations involve tumours or growths of various cellular origin. These lesions may include choristomas, hamartomas, hyperplasia, and harmless or dangerous neoplasms. A hamartoma is a benign focal malformation of tissue at the site of origin that lacks specific distinguishing features and is often designated as a neoplasm without consideration of pathology or biological behaviour. Here, we discuss the case of a 56-year-old man who presented with gradual dysphagia for 4 months along with sleep apnoea and voice changes. No palpable neck masses or abnormal lymphadenopathy was found. A left-sided homogeneous cyst-like mass was seen in the piriform fossa on laryngoscopy. The vocal cords were visualized and mobile. A contrast-enhanced computed tomography scan showed the obliteration of the piriform sinus on the left side by a faintly enhanced heterogeneous soft tissue lesion. The lesion originated from the left aryepiglottic fold, and was approached via micro-laryngoscopy and excised. Histopathological analysis led to a diagnosis of lipomatous hamartoma.

## الملخص

تظهر معظم أمراض البلعوم السفلية على شكل اورام أو نمو من أصول خلوية مختلفة. هذه الأورام قد تشمل المشيمية، الدموية، المتضخمة، والأورام غير الضارة أو الخطيرة. يعتبر الورم الدموي تشوه بؤري حميد في الأنسجة مكان نشأتها. تفتقر عادة الى سمات مميزة محددة وغالبا ما يتم تصنيفها على انها اورام دون النظر في خصائصها المرضية او سلوكها البيولوجي. هنا، نناقش حالة رجل يبلغ من العمر ٥٦- عاما والذي تعرض لعسر بلع تدريجي لمدة أربعة أشهر مع توقف النفس اثناء النوم وتغير في الصوت. لم يكن هناك ورم واضح في الرقبة أو تضخم غير طبيعي في العقد اللمفاوية. شوهد كيس متجانس على الجانب- الأيسر مثل -الكتلة بواسطة منظار الحنجرة في الحفرة الكمثرية. كانت الحبال الصوتية ظاهرة ومتحركة. أظهر التصوير المقطعي المحوسب- المحسن انسدادا في الجيب الكمثري على الجانب الأيسر من خلال تعزيز ضعيف لورم الأنسجة الرخوة غير المتجانسة. نشأ الورم من الطية الليفية اليسرى وتم استئصاله عن طريق تنظير الحنجرة- المجهري. أدى تحليل الأنسجة الى تشخيص الورم الدموي الشحمي

## Introduction

Hamartomas are tumour-like malformations composed of tissue elements specific to the organs in which they occur. The word hamartoma is derived from “hamartia,” meaning error, and it indicates a defect and local developmental disorder rather than a true neoplasm.^1^

Head and neck hamartomas are rare but have been found in the sinuses, nasopharynx, oral cavity, oropharynx, larynx, hypopharynx, cervical oesophagus, ears, parotid glands, trachea, parathyroid glands, and eyes. Hypopharyngeal hamartomas are extremely rare lesions, with very few cases documented in the medical literature. Additionally, their pathology is not well explained, or histological documentation is lacking.^2^ Patterson et al. have reported the first histologically confirmed hamartoma of the hypopharynx in the English literature.^1^

Head and neck hamartomas can markedly differ in appearance depending on their location. However, laryngeal and pharyngeal hamartomas may be detected incidentally in asymptomatic patients, or may cause dysphagia, hoarseness of voice, and stridor when large.^3^

The management of head and neck hamartomas depends on their location. However, hypopharyngeal hamartomas are usually adequately treated through excision via micro-laryngoscopy. Open surgery is rarely required for large lesions.^4^ The prognosis for completely resected lesions is very good.^2^ However, relapses are common, owing to inadequate resection. Even with inadequate resection, the recurrence rate is low.

Hamartomas of the hypopharynx are very rare. To better understand this disease, we report a case of hypopharyngeal hamartoma in a 56-year-old man who presented with dysphagia, changes in voice, and apnoea. The case is noteworthy because a provisional preoperative diagnosis could be made only through imaging, and led us to proceed with surgery through a conservative transoral route rather than an external approach.

## Case presentation

A 56-year-old man who was an ex-smoker with a history of diabetes mellitus presented to our clinic with dysphagia to solid foods that had gradually increased in the prior 4 months. The dysphagia was accompanied by changes in voice, difficulty in breathing, and apnoea that was more prominent when the patient lay on his right side; he was more comfortable when lying on his left side or back. The patient underwent a thorough clinical examination, and a flexible fibre optic laryngeal examination indicated a smooth-surfaced polypoid mass originating from the left piriform fossa in the hypopharynx. His vocal cords were visualized and mobile.

**Figure 1:**
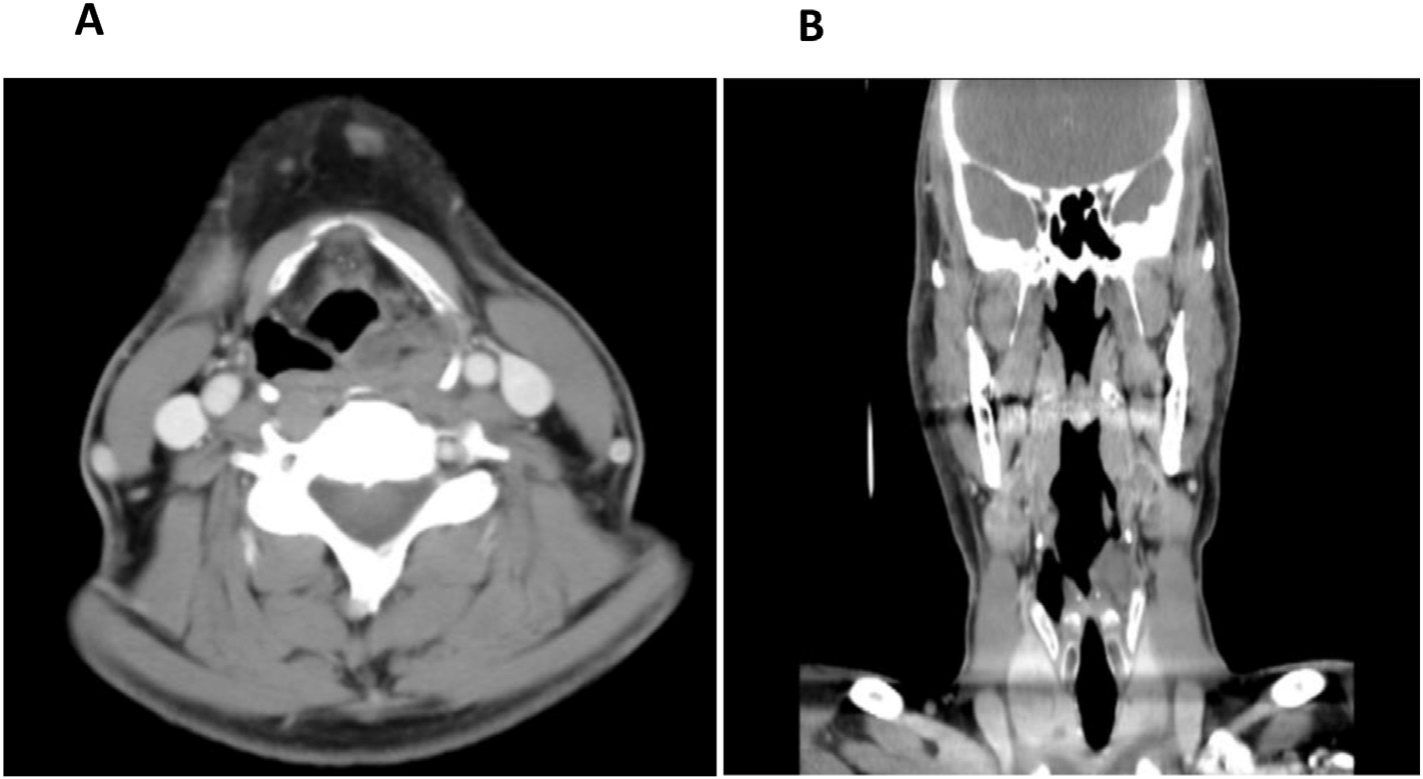
Computed tomography scan with contrast, showing obliteration of the piriform sinus on the left side with a faintly enhanced heterogeneous soft tissue lesion.

**Figure 2:**
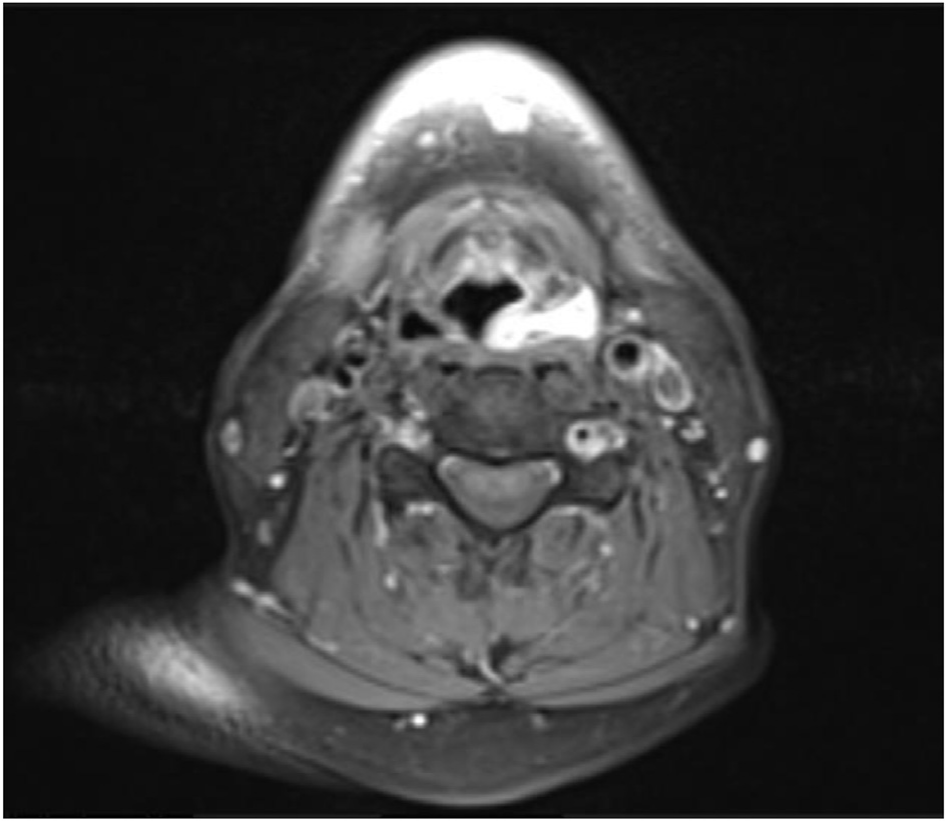
Magnetic resonance imaging with contrast of the left piriform fossa, showing a hypointense T2 signal with intense post-contrast enhancement.

A computed tomography scan with contrast showed obliteration of the piriform sinus on the left side by a faintly enhanced heterogeneous soft tissue lesion measuring 2.5 × 2.4 × 1.2 cm in craniocaudal view, on the basis of transverse and anterior-posterior diameters (Figure 1). There was no suspicion of associated lymphadenopathy. On magnetic resonance imaging with contrast, the left piriform fossa showed isointense T1 signal and hypointense T2 signal with intense post-contrast enhancement measuring approximately 2.4 × 2.5 × 1.3 cm in its largest dimensions (Figure 2).

**Figure 3:**
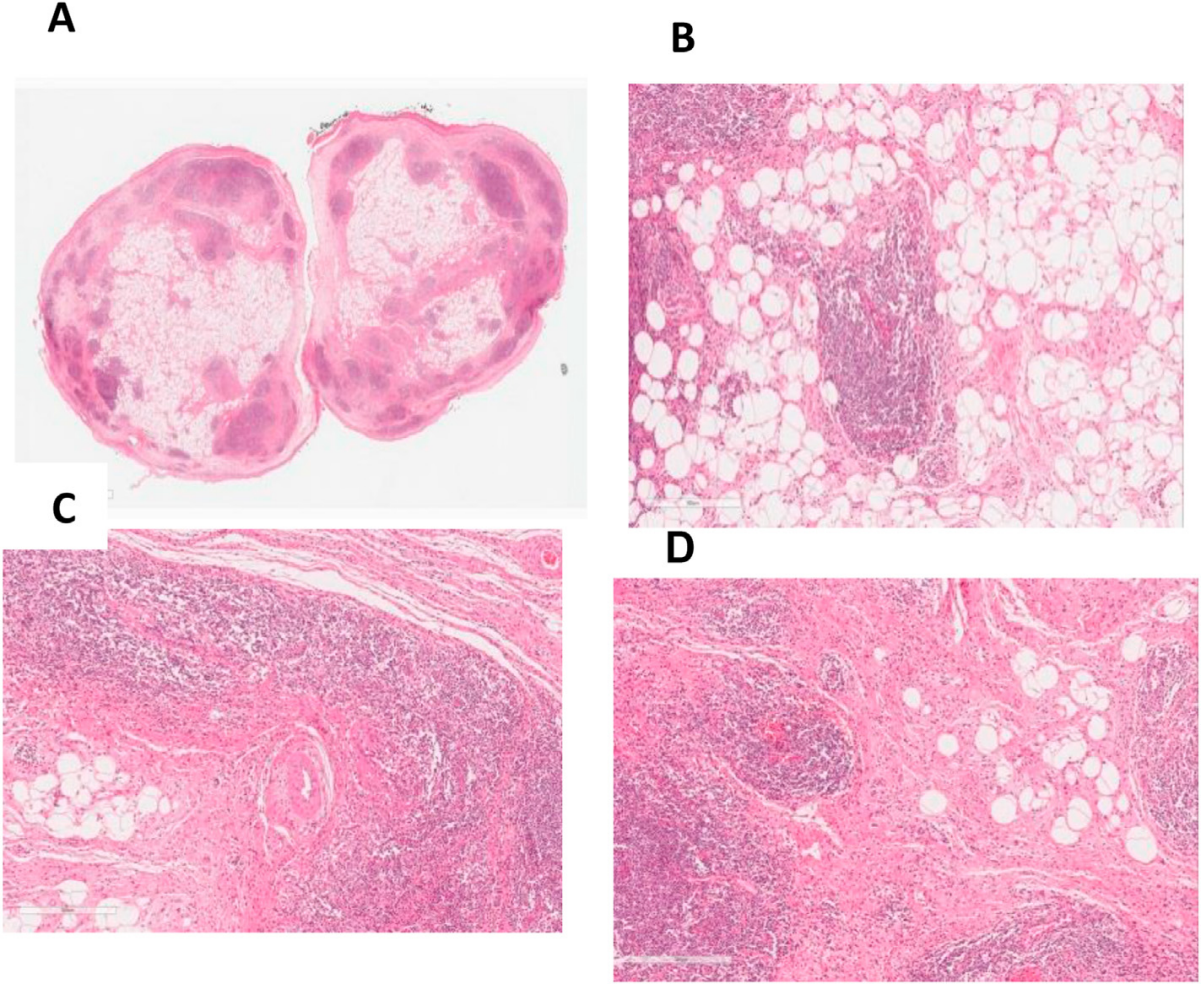
Microscopic features of our case of lipomatous hamartoma. (A): Polypoidal mass (panoramic view, formed by a mixture of mature fatty tissue, fibrous tissue, and benign lymphoid tissue forming lymphoid follicles with germinal centres in areas. (B, C, D): Haematoxylin and eosin staining at 10× magnification.

The patient underwent micro-laryngeal surgery to excise the left piriform fossa mass under general anaesthesia, and specimens were sent for microscopic analysis. Histological examination showed a non-encapsulated lesion measuring 1 cm in its largest dimension, formed from a mixture of mature fatty tissue, fibrous tissue, and benign lymphoid tissue, with formation of lymphoid follicles within germinal centres. On the basis of the microscopic findings, a diagnosis of lipomatous hamartoma was made (Figure 3).

**Figure 4:**
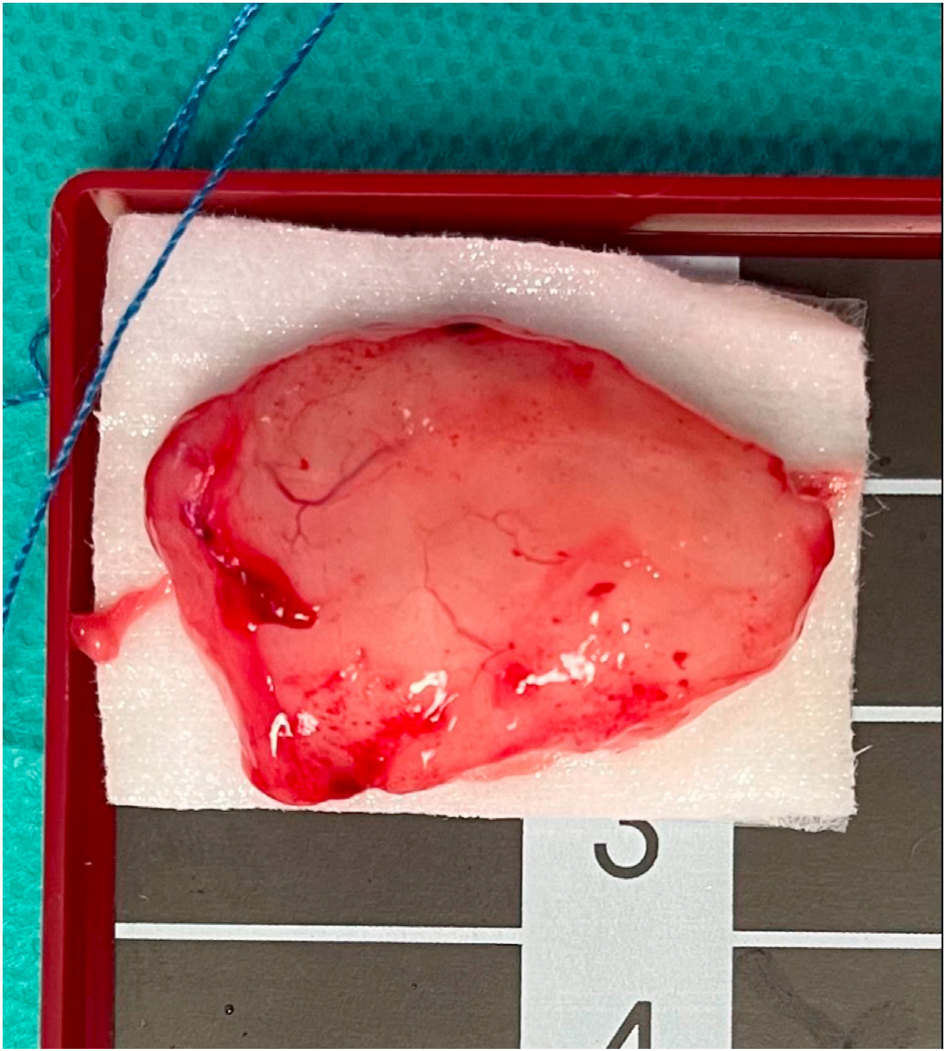
The surgical specimen, showing a non-encapsulated lesion measuring 2 × 2 × 3 cm, formed from a mixture of mature fatty tissue, fibrous tissue, and benign lymphoid tissue.

The patient improved after surgery. In the next follow-up visit after 6 months, the patient presented with a foreign body sensation in his throat and recurrent voice changes. On examination, there was no palpable mass in the neck. Repeat laryngoscopy showed mobile vocal cords and a left piriform cystic swelling prolapsing on the glottis (left false vocal cord mass). A second surgery was performed, and the mass was excised completely and was found to measure almost 5 cm in the largest dimension. Four months later, the patient appeared to be doing well and reported no changes in voice, dysphagia, or difficulty in breathing. Laryngoscopy examination indicated a normal-looking hypopharynx and larynx. Three years later, he presented again with the same prior complaint of a foreign body sensation in his throat and voice changes. On examination with laryngoscopy, a recurrent left-sided mass was seen in the piriform fossa; the rest of the examination was unremarkable. A third surgery was performed, and the recurrent left piriform fossa mass was excised. Its microscopic characteristics were similar to those of the previous specimens. The mass measured approximately 3 cm and was excised completely. The gross surgical specimen is shown in Figure 4.

## Discussion

We present a rare case of lipomatous hamartoma in a 56-year-old man with complaints of dysphagia and dyspnoea due to a piriform fossa mass, as confirmed by histopathology findings. Hamartoma of the hypopharynx is an extremely rare, non-malignant non-neoplastic tumour: only ten cases with published histology have been reported in children and adults in the literature.^3^^,^^5^, ^6^, ^7^, ^8^, ^9^ Although some hamartomas may be overlooked because of an absence of any symptoms, large hamartomas can cause life-threatening respiratory distress. Our patient had positional dyspnoea when the hamartoma closed the glottis in the right lateral recumbent positions. Other clinical signs and symptoms can include choking, wheezing, or inspiratory stridor. Swallowing-associated symptoms can include dysphagia and foreign body sensation during swallowing.^10^ Our case presented a mucosal fold causing a foreign body sensation in the throat with mild dysphagia.

Several investigative modalities are used to examine tumours in the larynx. Although clinical examination is usually performed first, it can be limited by the position and size of the mass. Laryngoscopy can aid in examination of lesions. Simple radiography is less helpful in characterizing lesions but may show a lobulated mass in as many as 50% of patients.^10^ Radiological studies, such as computed tomography or magnetic resonance imaging with contrast, can aid in diagnosis of most hamartoma and eliminate the need for prior lesion biopsy.^9^^,^^11^

Lipomatous hamartoma in the hypopharynx has been found to have better prognosis than hamartomas in other parts of the body.^12^ Recommendations for management of hypopharyngeal masses vary depending on their histopathology and sizes. In the case of a non-neoplastic mass, as in our case, patients can be placed under observation, or surgical excision can be performed, depending on patient performance. In the case of surgical excision, clear margins are important to minimize any chance of recurrence^13^ but might not be present in every case because of the location of the particular hamartoma. However, in our case, although the hamartoma was easily approached and excised through micro-laryngoscopy, relapse occurred, probably because of incomplete tumour resection. Recurrence rates have not been well described in the worldwide literature to date, particularly for hypopharyngeal hamartoma. However, 20% of partial resection of cases of laryngeal hamartoma have been estimated to relapse.^14^ Thus, regular follow-up with laryngoscopy in clinical settings should be arranged to detect any recurrence.

Notably, our patient had diabetes and was an ex-smoker. Nevertheless, owing to a lack of data, we found no association between diabetes or former smoking and head and neck hamartomas recurrence. Our patient underwent three surgeries within 5 years of presentation and recovered completely after each surgery. Recurrence of lipomatous hamartoma was suspected when he presented again with changes in voice and a globus sensation in the throat.

In our case, advances in imaging techniques allowed us to establish a preliminary diagnosis that led us to proceed transorally rather than following a more aggressive approach via lateral pharyngotomy, as described by Patterson et al.^1^ Hamartomas of the hypopharynx, although not considered high in the differential diagnosis of dysphagia, should nevertheless be kept in mind during detailed clinical and radiological work-up.^9^

## Conclusion

We report a unique case of a lipomatous hamartoma in the hypopharyngeal space that presented with dysphagia and dyspnoea. Advances in imaging techniques allowed us to establish a preliminary diagnosis and eliminate the need for a prior lesion biopsy. Histopathological studies confirmed the diagnosis. Subsequent follow-up and vigilance are necessary in such cases because of their rare nature and the importance of the location of occurrence. Inadequate resection and recurrence are common, but the likelihood of a complete recovery after recurrence is high.

## Source of funding

This research did not receive any specific grant from funding agencies in the public, commercial, or not-for-profit sectors.

## Conflict of interest

The authors have no conflicts of interest to declare.

## Ethical approval

Ethics approval was exempted by the institutional review board of King Abdullah International Medical Research Center (KAIMRC), Riyadh, KSA. [Date of approval 1 October 2019, reference number IRBC/1692/19].The need for informed consent was waived, because no information identifying an individual person was included.

## Consent

The need for informed consent was waived, because no information identifying an individual person was included.

## Authors contributions

KAA and BAA wrote initial draft of the article. AAA conceptualized the work, supervised, and wrote the final draft of the article. ALK contributed to the histopathology description. MAA contributed supervision, revised the article, and approved the final version. All authors have read and approved the final manuscript. All authors have critically reviewed and approved the final draft and are responsible for the content and similarity index of the manuscript.
